# Genetically Predicted Iron Status Is a Causal Risk of Rheumatoid Arthritis: A Mendelian Randomization Study

**DOI:** 10.1055/s-0044-1789259

**Published:** 2024-08-29

**Authors:** Boyuan Wu

**Affiliations:** 1 School of Global Public Health New York University, New York, New York, United States

**Keywords:** iron, ferritin, rheumatoid arthritis, Mendelian randomization, single nucleotide polymorphism

## Abstract

**Background** Current knowledge on iron's role in rheumatoid arthritis (RA) development is very limited, with studies yielding inconsistent findings. We conducted a two-sample Mendelian randomization study to assess the associations of iron status with the risk of RA.

**Methods** This study leveraged genetic data from a large genome-wide association study (GWAS) of 257,953 individuals to identify single nucleotide polymorphisms (SNPs) associated with iron status. We then analyzed these data in conjunction with summary-level data on RA from the IEU open GWAS project, which included 5,427 RA cases and 479,171 controls. An inverse-variance weighted method with random effects was employed, along with sensitivity analyses, to assess the relationship between iron status and RA risk.

**Results** Genetic predisposition to high ferritin and serum iron status was causally associated with lower odds of RA. Ferritin had an odds ratio (OR) of 0.997 (95% confidence interval [CI]: 0.995–0.997; *p* = 0.010), indicating that a one-unit increase in ferritin is associated with a 0.3% decrease in the odds of RA. Similarly, serum iron had an OR of 0.997 (95% CI: 0.995–0.999; *p* = 0.014). However, MR analyses found no significant causal associations between total iron-binding capacity (OR = 1.0, 95% CI: 0.999–1.002; *p* = 0.592) or transferrin saturation percentage (OR = 0.998, 95% CI: 0.996–1.000; *p* = 0.080) and risk of developing RA.

**Conclusions** This study suggests that individuals with genes linked to higher iron levels may have a lower risk of developing RA. Our findings indicate that the total amount of iron in the body, rather than how it is distributed, might be more important for RA. This raises the intriguing possibility that iron supplementation could be a preventative strategy, but further research is necessary.

## Introduction

Rheumatoid arthritis (RA) is a common systemic autoimmune and inflammatory disease that is predominantly featured by synovial inflammation. It stands as the most prevalent form of inflammatory arthritis. The incidence of RA is approximately 0.5 to 1%, with a male-to-female ratio of 2.5/1. It typically affects individuals between the ages of 40 and 70, with the incidence increasing with age. Tragically, approximately 40% of patients with RA become disabled after 10 years. 1

RA attacks the synovial cells and chondrocytes in joints, causing inflammation in the synovium, damage to cartilage, and even bone erosion. As the disease progresses, local changes in the synovial tissue lead to chronic, symmetrical inflammation in multiple joints (polyarthritis) and may involve tissues beyond the joints (extra-articular lesions). 2 RA primarily affects small joints, like those in the hands, wrists, and feet, with recurring flare-ups and symmetrical symptoms. Common clinical symptoms include joint pain, swelling, and stiffness, which can worsen over time and lead to joint destruction and disability. 1 This significantly impacts a patient's functionality and quality of life.

RA currently lacks a definitive cure. Treatment focuses on managing symptoms and slowing disease progression. 2 Further research is crucial to improve prevention, diagnosis, and treatment options for this debilitating condition.

While the pathogenesis of RA remains not fully understood, several risk factors are established, including gender, smoking status, obesity, Graves' disease, and several genetic alterations. 2

Micronutrients are essential for maintaining homeostasis and are linked to many diseases, including RA. Iron, a key component of hemoglobin and myoglobin, is critical for transporting oxygen throughout the body, storing it in muscles, and enabling cells to utilize it for energy production. It also plays a vital role within mitochondria by helping transfer electrons in the energy-generating electron transport chain. In addition, iron is an essential trace element critical for various biological processes, 3 playing a central role in maintaining human health. Balanced iron levels are crucial, as both deficiency and overload are associated with numerous diseases, impacting immune function and inflammatory responses. 4 5 Tightly regulated iron homeostasis ensures optimal health. Recent advancements have shed light on iron's role in modulating immune cell function and its connection to various human diseases. 6 For instance, intracellular iron in neuroinflammatory diseases appears to drive the differentiation of pathogenic Th cells by promoting the production of the proinflammatory cytokine GM-CSF. 7 Conversely, iron deficiency hinders B cell proliferation and antibody responses, highlighting its potential role in humoral immunity and vaccination efficacy. 8 Given RA is characterized as an autoimmune disease, and taking into account the facts that the blood iron levels in patients with RA were significantly lower than the control and negatively correlated with disease activity, 9 we hypothesize that abnormal iron status might be a causal risk factor for RA. However, current knowledge on iron's role in RA development remains limited, with studies yielding inconsistent findings. 1 2 10 11 12

Mendelian randomization (MR) study offers a powerful approach to estimate the causal effect of an exposure on a disease outcome. It leverages genetic variants as instrumental variables (IVs) for the exposure. Under specific assumptions, MR can isolate the causal effect by effectively bypassing confounding variables that might distort traditional observational studies. These assumptions center around the IVs: (1) they must be robustly associated with the exposure, (2) they should have no independent association with factors that confound the exposure–outcome relationship, and (3) their influence on the outcome must solely operate through the exposure (given confounders are absent). An additional assumption, monotonicity, is often required to definitively establish causality.

The potential for a two-way causal relationship between iron deficiency and RA necessitates a robust approach to untangle their association. MR is ideally suited for this purpose. This study will leverage MR to investigate whether iron deficiency causally influences the risk of developing RA.

## Methods

### MR Study Design

**Fig. 1:**
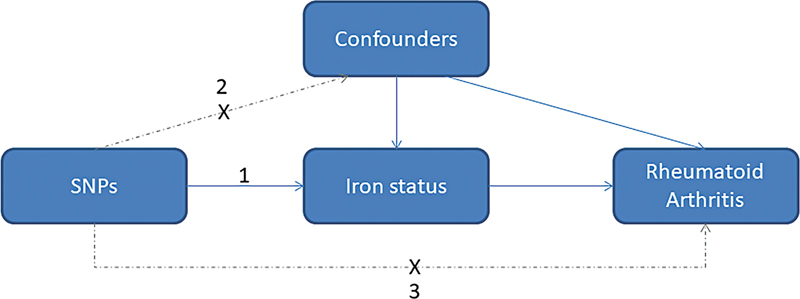
The causal-directed acyclic graph of the two-sample Mendelian randomization study. Three hypotheses of MR study are that (1) instrumental variables (SNPs, single nucleotide polymorphisms) are strongly associated with the iron status, including ferritin, serum iron, total iron-binding capacity, and transferrin saturation; (2) they are not associated with any potential confounders; and (3) they do not affect rheumatoid arthritis independent of the iron status.

We performed a two-sample MR study to investigate the potential causal associations of four sets of IVs regarding iron status with the risks of RA. Three hypotheses of our MR study are that (1) genetic IVs are strongly associated with the iron status, including ferritin, serum iron, total iron-binding capacity (TIBC), and transferrin saturation percentage (TSP); (2) they are not associated with any potential confounders; and (3) they do not affect RA independent of the iron status ( Fig. 1 ). There is no need to pre-register the protocol.

### Data Source and Software

The work presented was performed using publicly available summary-level data from published genome-wide association study (GWAS).

Iron status data were sourced from the largest available GWAS on iron traits. 13 14 This GWAS is a meta-analysis of studies conducted in six European populations (DeCODE, INTERNAL, SardiNIA, DBDS, HUNT, and MGI). It analyzed four iron status biomarkers: serum iron ( *n* = 236,612), TSP ( *n* = 198,516), ferritin ( *n* = 257,953), and TIBC ( *n* = 208,422). The GWAS datasets are publicly available for download through NTNU Open Research Data ( https://doi.org/10.18710/S9TJEL ).

RA outcome data were retrieved from the IEU open GWAS project, specifically GWAS ID “ebi-a-GCST90038685” ( https://gwas.mrcieu.ac.uk ). This dataset comprises 5,427 RA cases and 479,171 controls.

All analyses were performed using R version 4.3.2 (R Foundation for Statistical Computing, Vienna, Austria). While the MR analysis code is available upon reasonable request from the corresponding author, institutional review board approval was not required because this study utilized publicly available summary statistics and did not involve any patient interaction.

### Instrumental Variable Selection

To ensure the robustness of the MR analysis results, we implemented a rigorous quality control protocol for instrument variable (IV) selection. First, strong associations between the IVs and exposure were established. Single nucleotide polymorphisms (SNPs) significantly associated with each iron trait at a genome-wide level ( *p* < 5 × 10 ^−8^ ) were extracted from the iron GWAS meta-analysis. Second, to minimize the influence of linkage disequilibrium (LD) on the analysis, we excluded SNPs in high LD (defined by *r* ^2^ < 0.001 and clump distance > 10,000 kb). This ensured independent effects of the selected IVs. Third, we excluded SNPs directly associated with the outcome ( *p* < 5 × 10 ^−6^ ) to minimize potential pleiotropic effects. Finally, we controlled for confounding factors potentially influencing the MR analysis, such as smoking and obesity, by removing them from the PhenoScanner database (http://www.phenoscanner.medschl.cam.ac.uk). Palindromic SNPs with intermediate allele frequencies were excluded. Proxies were used to replace the missing SNPs in the outcome dataset.

### Statistical Analysis

A two-sample MR analysis was performed using the “TwoSampleMR” package to investigate the causal relationship between iron status and RA development. The random-effects inverse-variance weighted (IVW) method served as the primary analysis due to its accuracy when IV assumptions hold. Additionally, several complementary methods were employed for robustness: MR-Egger to detect and adjust for pleiotropic effects (though with potential for lower precision, indicated by a *p* -value for intercept <0.05), weighted median for accuracy assuming at least half the IVs are valid, and simple/weighted mode for alternative estimates. The lower power of these complementary methods compared to IVW necessitated prioritizing the IVW findings for interpretation.

**Fig. 2:**
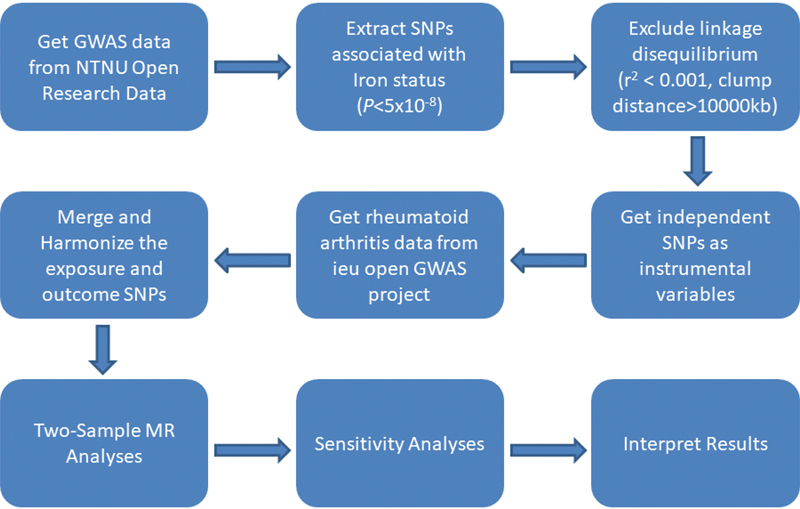
Mendelian randomization study flowchart. The boxes represent research steps and the arrows indicate the flow direction. GWAS, genome-wide association study; MR, Mendelian randomization; SNP, single nucleotide polymorphism.

To assess potential heterogeneity, which could bias the results, we employed the MR-heterogeneity assay. Funnel plots were also generated to visually inspect for heterogeneity. MR-PRESSO and leave-one-out tests were used to identify and remove outliers in the MR analysis. Additionally, the intercept term of the MR-Egger method was analyzed ( *p* -value < 0.05 for significance) to identify pleiotropic effects, where SNPs influence both exposure and other traits besides the outcome. The flowchart of this study is presented in Fig. 2 .

## Results

### IV Selection

Following stringent quality control, we identified SNPs significantly associated with ferritin, serum iron, TIBC, and TSP. We then excluded SNPs in LD to avoid redundancy. This resulted in 51 ferritin-associated SNPs, 25 iron-associated SNPs, 34 TIBC-associated SNPs, and 25 TSP-associated SNPs. Importantly, none of these SNPs showed association with the outcome or potential confounders. Finally, we identified and removed outliers: rs114355928 from ferritin, rs9273076 from TIBC, and rs185520326 from TSP.

### MR Analysis

**Fig. 3:**
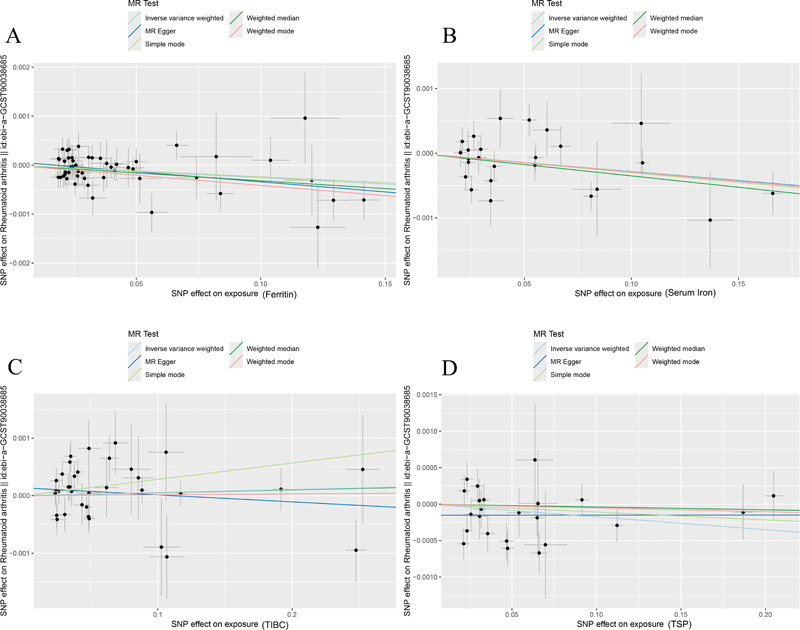
Scatter plots visualize the causal association between genetically predicted iron status and rheumatoid arthritis using five Mendelian randomization (MR) methods. Each data point represents an instrumental variable (IV), with its horizontal position reflecting the SNP effect on an iron status measure: ( **A** ) ferritin, ( **B** ) serum iron, ( **C** ) TIBC, and ( **D** ) TSP. The vertical position shows the SNP effect on rheumatoid arthritis. Lines connect the SNP effects for each MR method: inverse-variance weighted (light blue), MR-Egger (dark blue), simple mode (light green), weighted median (dark green), and weighted mode (pink). The line's slope represents the causal estimate. A negative slope suggests an inverse effect of iron status on rheumatoid arthritis. MR, Mendelian randomization; SNP, single nucleotide polymorphism; TIBC, total iron-binding capacity; TSP, transferrin saturation percentage.

The MR scatter plot and random-effects IVW analysis provided strong evidence supporting the ferritin and serum iron were significantly inversely associated with RA development. Ferritin had an odds ratio (OR) of 0.997 (95% confidence interval [CI]: 0.995–0.997; *p* = 0.010), indicating that a one-unit increase in ferritin is associated with a 0.3% decrease in the odds of RA. Similarly, serum iron had an OR of 0.997 (95% CI: 0.995–0.999; *p* = 0.014). The results of complementary analyses (MR-Egger, weighted median, simple mode, and weighted mode) showed the same trend as IVW. On the other hand, the IVW and other analyses found no significant causal associations between TIBC (OR = 1.0, 95% CI: 0.999–1.002; *p* = 0.592) or TSP (OR = 0.998, 95% CI: 0.996–1.000; *p* = 0.080) and risk of developing RA ( Fig. 3 ).

**Fig. 4:**
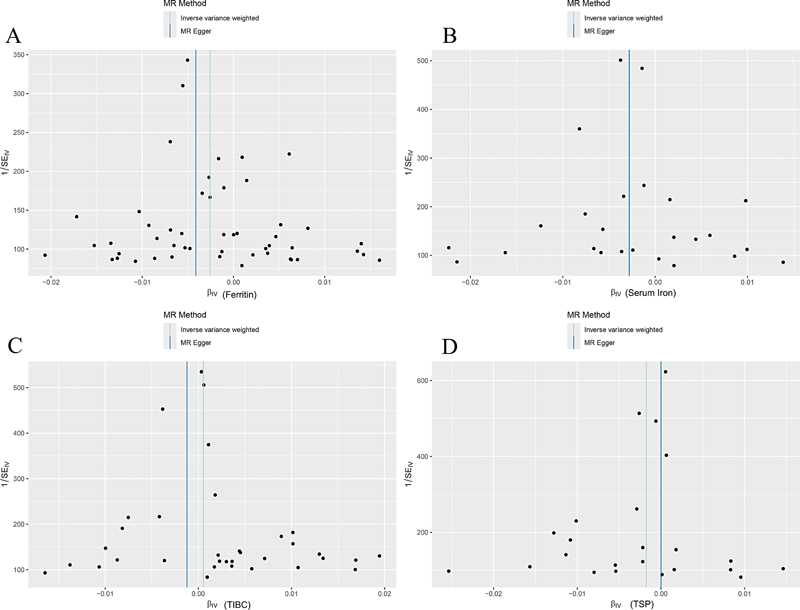
The funnel plots of the causality of iron status and rheumatoid arthritis were symmetrically distributed for ( **A** ) ferritin, ( **B** ) serum iron, ( **C** ) TIBC, but not ( **D** ) TSP. TIBC, total iron-binding capacity; TSP, transferrin saturation percentage.

**Fig. 5:**
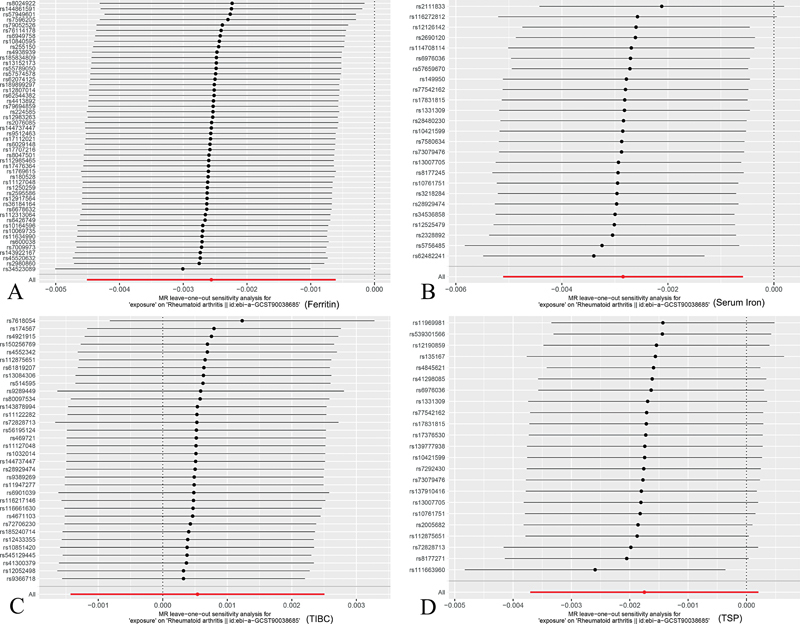
The leave-one-out graph indicated that removal of any SNPs had no fundamental effect on the results, suggesting that the MR results were reliable for ( **A** ) ferritin, ( **B** ) serum iron, ( **C** ) TIBC, and ( **D** ) TSP. MR, Mendelian randomization; TIBC, total iron-binding capacity; TSP, transferrin saturation percentage.

Our analyses revealed no significant evidence of heterogeneity in the causal estimates for ferritin ( *p* = 0.706), serum iron ( *p* = 0.076), or TSP ( *p* = 0.081). Additionally, symmetrical funnel ( Fig. 4 ) plots for ferritin, iron, and TSP suggested minimal publication bias. Furthermore, the leave-one-out analysis ( Fig. 5 ) confirmed that no single SNP significantly influenced the overall association between iron status and RA. Finally, MR-Egger intercept analysis detected no pleiotropic effects for any of the investigated exposures: ferritin ( *p* = 0.320), serum iron ( *p* = 0.974), TIBC ( *p* = 0.178), or TSP ( *p* = 0.155).

## Discussions

Emerging studies reveal a complex interplay between iron metabolism and autoimmune diseases. In neuroinflammatory conditions, intracellular iron appears to fuel the development of pathogenic Th cells by promoting GM-CSF production. 7 Conversely, iron deficiency hinders B cell proliferation and antibody responses, highlighting its role in regulating humoral immunity and potentially impacting vaccination efficacy. 8 Recent findings link the production of tetrahydrobiopterin (BH4) in activated T cells to changes in iron metabolism and mitochondrial function. Blocking BH4 synthesis improved outcomes in T cell-mediated autoimmunity and allergic inflammation, suggesting a promising therapeutic target. 15 Collectively, these studies underscore the critical role of iron metabolism in T cell function and autoimmune disease development. Furthermore, a recent study 16 suggests that iron overload can exacerbate the differentiation of Tfh and Th1/Th17 cells, ultimately promoting antibody production and autoimmune response in systemic lupus erythematosus. The findings solidify the established importance of iron in autoimmune disorders.

Given the chronic autoimmune nature of RA, it is reasonable to hypothesize that iron metabolism plays a role in its pathogenesis. However, existing studies on RA present conflicting data, necessitating further investigation. For example, in a prospective cohort study consisting of 546 cases of incident RA among 82,063 women, 1 the multivariate models revealed no association between RA and any measure of iron or protein intake, including red meat, poultry, and fish. While a two-sample MR study 11 suggested a protective effect of genetically determined high iron status against RA, this finding was not replicated in subsequent studies. Two additional MR studies 10 12 and a meta-analysis 2 did not observe an association between iron-related SNPs and RA development.

It is generally accepted that RA often leads to iron deficiency and anemia, which can worsen physical disability and increase mortality. 17 18 Iron deficiency was found in 64% of RA patients, while for women it was 76%. Thus, iron deficiency was very common among RA patients and its prevalence was several times higher than the prevalence of anemia. Iron deficiency can be absolute or functional. 19 Absolute iron deficiency occurs when the body's iron stores are depleted, often due to insufficient dietary iron intake, impaired iron absorption in the gut, or chronic blood loss, typically from the gastrointestinal tract. Functional iron deficiency, on the other hand, arises when iron is available but not readily usable by cellular processes. 19 When we explore the question if iron status is a causal risk of developing RA, the above-mentioned facts constitute a reverse causation and complicate the study.

MR is a powerful technique that leverages genetic IVs as proxies to investigate causal relationships between environmental exposures and health outcomes. Since these genetic variants are randomly assigned at conception, they are less susceptible to confounding factors like lifestyle, obesity, or environmental influences that might otherwise distort the true cause-and-effect relationship. MR studies, when equipped with sufficient sample sizes and carefully chosen SNPs, offer a significant advantage by mitigating the influence of reverse causation, potentially leading to more definitive conclusions.

Our study identified a potential protective effect of genetically predicted higher ferritin and serum iron levels against RA. This finding contrasts with previous MR studies 10 12 that did not observe an association between iron-related SNPs and RA development. The discrepancy likely stems from methodological differences. While prior studies used a limited number of IVs (3–11 SNPs) in samples of less than 50,000 individuals, our analysis leveraged a larger dataset (>250,000 individuals) and a more comprehensive set of IVs (50 ferritin-associated SNPs and 25 iron-associated SNPs). This allowed us to detect a more subtle causal relationship between ferritin/iron levels and RA development.

Ferritin, a protein found throughout the body, acts as a cellular iron bank. It safely stores iron and releases it when needed. In contrast, serum iron refers to the iron circulating in the bloodstream, readily available for functions like red blood cell production. Together, ferritin and serum iron represent most of the body's iron stores. While TIBC and TSP reflect the body's capacity to transport and distribute iron, our findings suggest that the total amount of iron rather than its distribution plays a role in RA. This raises the possibility that iron supplementation could be a strategy for RA prevention, but further research is needed. To strengthen our findings and establish a better causal link, we propose conducting prospective cohort studies, which could monitor ferritin and iron levels, track long-term iron supplementation, and assess the incidence of RA. This approach would provide more compelling evidence regarding the potential role of iron in RA prevention.

## Conclusion

This MR study suggests that individuals with genes linked to higher iron levels may have a lower risk of developing RA. Our findings indicate that the total amount of iron in the body, rather than how it is distributed within tissues, might be more important for RA. This raises the intriguing possibility that iron supplementation could be a preventative strategy, but further research is necessary before making any recommendations.
